# Metabolic reprogramming during the *Trypanosoma brucei *life cycle

**DOI:** 10.12688/f1000research.10342.2

**Published:** 2017-05-18

**Authors:** Terry K. Smith, Frédéric Bringaud, Derek P. Nolan, Luisa M. Figueiredo

**Affiliations:** 1 Biomedical Sciences Research Complex, University of St Andrews, Fife, UK; 2 Laboratoire de Microbiologie Fondamentale et Pathogénicité (MFP), UMR 5234 CNRS, Université de Bordeaux, Bordeaux, France; 3 School of Biochemistry and Immunology, Trinity Biomedical Sciences Institute, Trinity College Dublin, Dublin, Ireland; 4 Instituto de Medicina Molecular, Faculdade de Medicina, Universidade de Lisboa, Lisboa, Portugal

**Keywords:** Trypanosoma brucei, metabolism, adaptations

## Abstract

Cellular metabolic activity is a highly complex, dynamic, regulated process that is influenced by numerous factors, including extracellular environmental signals, nutrient availability and the physiological and developmental status of the cell. The causative agent of sleeping sickness, *Trypanosoma brucei*, is an exclusively extracellular protozoan parasite that encounters very different extracellular environments during its life cycle within the mammalian host and tsetse fly insect vector. In order to meet these challenges, there are significant alterations in the major energetic and metabolic pathways of these highly adaptable parasites. This review highlights some of these metabolic changes in this early divergent eukaryotic model organism.

## 1. Introduction

*Trypanosoma brucei* is a unicellular protozoan parasite, transmitted by the bite of tsetse flies ( *Glossina* genus). Different species/subspecies of trypanosomes infect a variety of different vertebrates, including animals and humans. Human African trypanosomiasis (HAT), also known as sleeping sickness, is caused by two subspecies: *Trypanosoma brucei gambiense* and *Trypanosoma brucei rhodesiense*. In recent years, the number of reported cases of HAT has decreased steadily, falling to just about 6,000 in 2013 ^1^. Other trypanosome species infect both domestic and wild animals, causing animal African trypanosomiasis. The infection of livestock has a major impact on the African economy, limiting the production of milk and meat and the development of agriculture in areas otherwise amenable to animal husbandry ^2^.

Trypanosomatids are also of intrinsic scientific interest as they separated early (>600 million years ago) and have evolved differently from other well-studied eukaryotes ^3^. *T. brucei brucei* (here called *T. brucei*), a subspecies non-infectious to human, is by far the best characterised. In the mammalian host, *T. brucei* parasites colonise the blood and interstitial spaces of several tissues, including the brain, adipose tissue and skin ^4– 6^. The presence of parasites in the brain is associated with the appearance of the sleep disorder and neurological symptoms characteristic of later stages of the disease ^1^.

**Figure 1.**
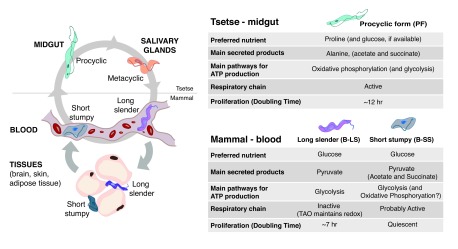
Changes in metabolism during the life cycle of *Trypanosoma brucei*. *T. brucei * life cycle spans two hosts: a mammal (human, cattle, wild animals) and the tsetse fly. As this protozoan parasite is extracellular, it adapts its metabolism to the available extracellular nutrients. The two stages that have been better characterised in terms of metabolism are the bloodstream long slender and procyclic forms, which mainly catabolise glucose and proline, respectively. Fewer studies have studied bloodstream short stumpy forms. In the mammalian host, parasites accumulate in the interstitial spaces of several tissues, mainly the brain, skin and visceral adipose tissue (adipocytes are shown as an example). The metabolism of parasites in these tissues remains mostly unknown, except for the activation of fatty acid β-oxidation in parasites resident of the adipose tissue. Metabolism of metacyclic stage has not been characterised to date. TAO, trypanosome alternative oxidase.

In the mammalian host, parasites exist in two stages: bloodstream long slender form (B-LS), which doubles every 7 hours by binary fission, and short stumpy form (B-SS), which is terminally cell cycle–arrested ( Figure 1). The differentiation from B-LS to B-SS is irreversible and is triggered by a quorum-sensing mechanism ^7^. The B-SS form is pre-adapted to life in the tsetse fly midgut ^7^. These pre-adaptions probably help in the efficient differentiation into the replicative procyclic forms (PFs). Eventually, PFs migrate from the midgut to the proventriculus, where they further differentiate into epimastigotes and later into metacyclics in the salivary glands ( Figure 1). The latter are cell cycle–arrested and are able to re-colonise/re-infect a mammalian host when a tsetse fly takes a blood meal.

Throughout the life cycle, parasites encounter and adapt to very different environments. In the mammalian host, such adaptations include avoidance of the host immune system (by employing antigenic variation) as well as metabolic adaptations to use available nutrients. For example, the brain glucose levels is normally 10–20% of blood levels ^8^, whereas adipose tissue may be a better source of lipids. In the tsetse fly vector, the parasites face a proteolytic rather than immune challenge and also have to adapt to an environment that is free of glucose but rich in amino acids, particularly proline ^9^. *T. brucei* re-programmes its metabolism in order to benefit from the nutrients available in the environment. In this review, we will compare the metabolic differences that take place during the *T. brucei* life cycle, highlighting the questions that remain unanswered. To facilitate the understanding of this review by a non-metabolism expert, we will first summarise the main metabolic pathways present in most eukaryotic cells.

## 2. Basics of eukaryote metabolism

### 2.1. Multiple carbon sources for energy production

All living organisms use adenosine triphosphate (ATP) as an intracellular energy source. ATP is generated by the catabolism (breakdown) of nutrients. The most common nutrients or carbon sources are carbohydrates (such as glucose), fatty acids and amino acids.

Most organisms derive energy from the breakdown of glucose, by a process known as glycolysis, a universal and evolutionarily ancient metabolic pathway, which converts glucose (6-carbon) into pyruvate (3-carbon). Under aerobic conditions, pyruvate can undergo further breakdown to acetyl coenzyme A (acetyl-CoA) (2-carbon) and subsequently to carbon dioxide (CO _2_) via the tricarboxylic acid (TCA) cycle with the concomitant production of reducing equivalence (NADH and FADH _2_) and GTP. Transfer of electrons from these reduced cofactors to oxygen via an electron transport chain generates a proton electrochemical gradient across the mitochondrial inner membrane that is used to generate ATP by a membrane-bound ATP synthase, collectively a process termed oxidative phosphorylation (OXPHOS). The complete oxidation of each glucose molecule leads to the production of about 36 ATP molecules, showing how OXPHOS is a very efficient mechanism of producing energy.

In the absence of oxygen, the glycolysis product (pyruvate or phosphoenolpyruvate) can be further metabolised by fermentation into excreted end-products, such as lactate (for instance, in humans during running) and ethanol (for instance, in yeast) in the cytoplasm, leading to the net production of two ATP molecules per molecule of glucose consumed. Although the flux through fermentation can be very high, the pathway is energetically inefficient in terms of ATP production. In the absence of oxygen, some microorganisms use nitrate ions, sulfate ions, and carbon dioxide as final electron acceptors, in a process named anaerobic respiration. For example, the final product of glycolysis could be converted into acetyl-CoA, which enters the TCA cycle or is converted into acetate. The electrons are then donated to the final acceptor through the mitochondrial electron transport chain.

In many organisms, fatty acids can be catabolised via β-oxidation in the mitochondria to again generate the 2-carbon unit acetyl-CoA, which feeds into the TCA cycle and OXPHOS. Fatty acid β-oxidation of a palmitate molecule (a fatty acid with 16 carbons that is very abundant in mammalian adipocytes) can generate 106 ATP molecules. The balance between making and breaking down fatty acids is tightly regulated.

Amino acids can also contribute to total energy production by oxidation to urea and CO _2_. The first reaction is the removal of the amino group by transaminases. While the amino group enters the urea cycle, the ketoacid carbon skeletons typically enter the TCA cycle and fuel OXPHOS.

The relative abundances of sugars, amino acids, and fatty acids along with the availability of sufficient oxygen to use OXPHOS influence which metabolic pathways are preferentially used to produce ATP. Thus, the metabolic profile of a cell is a consequence of the regulated expression of pathway-specific proteins and associated transporters in response to extracellular nutritional and environmental conditions ^10^.

### 2.2. Metabolic adaptations in eukaryotes

Textbooks on metabolism explain that in nutrient-rich conditions, model unicellular organisms undergoing exponential growth often use fermentation ^11^. Proliferating cells in a multicellular organism also metabolise glucose primarily through glycolysis, secreting ethanol, lactate, or another organic acid such as acetate. When unicellular organisms are starved of nutrients, they switch and rely primarily on oxidative metabolism, as do terminally differentiated cells in a multicellular organism. It is no surprise that there are many exceptions to these generalised concepts, and as we will describe below (Section 4), *T. brucei* is a quintessential example of these exceptions.

The metabolism of cells is a highly regulated process that is influenced by numerous extracellular factors. For example, yeast uses glucose from the environment as their preferred carbon source. Even in the presence of oxygen, glucose is converted into excreted ethanol, with a low yield of ATP production. Although this process may seem wasteful, it is a highly efficient way to support exponential growth. When glucose levels are low, yeast undergoes a diauxic switch to another carbon source, ethanol, which requires an alteration of its mitochondrial metabolism. By using this alternative carbon source, cells are able to continue to grow and divide, but at a significantly reduced rate ^12^.

In mammals, most non-proliferating differentiated cells use glycolysis and OXPHOS to generate ATP and convert glucose to CO _2_ and H _2_O. However, most proliferating cancer cells convert glucose into pyruvate and lactate (3-carbon) even under aerobic conditions, a phenomenon known as the Warburg effect, named after its discoverer ^11, 13^. Although this fermentation-like process is intrinsically energetically less efficient, these cells use far higher rates of glycolysis to meet their higher metabolite demand as they divide faster. This metabolic reprogramming allows cancer cells to rapidly produce the building blocks and increase total biomass for a faster propagation time ^14^.

Major metabolic changes also take place when immune cells are activated and initiate proliferation. When T cells are activated upon infection or inflammation, gene expression is reprogrammed, resulting in rapid growth, proliferation and the acquisition of new effector functions. Effector T cells, like cancer cells, rely upon aerobic glycolysis when proliferating ^11^. In contrast, T cells destined to become memory cells maintain an oxidative metabolism, which allows them to keep their quiescence and longevity. Regulatory T (Treg) cells predominantly use OXPHOS for development and survival ^15, 16^, while activated B cells show increased glucose uptake and induction of glycolysis ^17^. These examples demonstrate how malleable metabolism is, as cells react to environmental factors and signals to acquire new functions.

## 3. Trypanosomes have unusual metabolic features

### 3.1. Trypanosomes have a single mitochondrion and glycosomes

**Figure 2.**
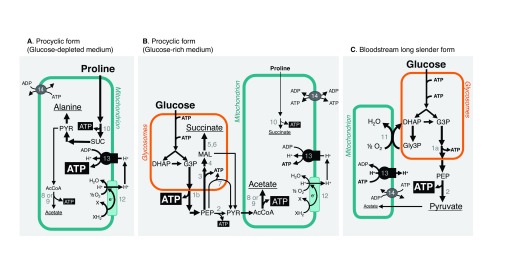
Multiple pathways to produce ATP in trypanosomes. Catabolism of the most abundant carbon sources in procyclic form grown in glucose-depleted ( **A**) or in glucose-containing ( **B**) conditions and in bloodstream long slender form ( **C**). Excreted end-products from glucose and proline degradation (pyruvate, acetate, succinate and alanine) are underlined. Arrows with different thicknesses tentatively represent the metabolic flux at each enzymatic step. In ( **B**), the direction of ADP/ATP exchange between the cytosol and the mitochondrion (step 14) is unknown and is represented by double arrows. Key enzymatic steps: 1a, glycosomal phosphoglycerate kinase; 1b, cytosolic phosphoglycerate kinase; 2, pyruvate kinase; 3, phosphoenolpyruvate carboxykinase; 4, glycosomal malate dehydrogenase; 5, cytosolic fumarase (for simplification this reaction is placed in the glycosome); 6, glycosomal NADH-dependent fumarate reductase; 7, pyruvate phosphate dikinase; 8, acetate:succinate coenzyme A-transferase, or ASCT; 9, acetyl-coenzyme A thioesterase; 10, succinyl-coenzyme A synthetase; 11, trypanosome alternative oxidase; 12, respiratory chain; 13, F _0_F _1_-ATP synthase; 14, mitochondrial ADP/ATP exchanger. AcCoA, acetyl-coenzyme A; DHAP, dihydroxyacetone phosphate; G3P, glyceraldehyde 3-phosphate; Gly3P, glycerol 3-phosphate; MAL, malate; PEP, phosphoenolpyruvate; PYR, pyruvate; SUC, succinate.

Trypanosomes are characterised by the presence of a dense network of circularised interlocking rings of mitochondrial DNA termed the kinetoplast, located within the large, single mitochondrion of the cell. The single mitochondrion in PFs has a highly defined branched structure with discoid cristae, whereas in the B-LS forms the organelle is a less well-developed narrow tubular structure with an acristate morphology similar to that of the promitochondrion of anaerobic yeast ^18, 19^. *T. brucei* also contains peroxisome-like organelles, named glycosomes, which contain the first six (PF) or seven (B-LS) glycolytic enzymes ^3^. Since the glycosomal membrane is impermeable to ATP, no net ATP production occurs inside these organelles. Thus, net ATP production from glycolysis occurs during the cytoplasmic steps ( Figure 2).

In contrast, ADP and ATP molecules can be exchanged between the cytosolic and mitochondrial compartments through the TbMCP5 mitochondrial ADP/ATP exchanger ^20^. This exchanger is required because oxidative phosphorylation does not occur in B-LS and thus no ATP is generated inside this organelle. To maintain the mitochondrial proton electrochemical gradient across the mitochondrial membrane, the F1F0-ATPase operates in the reverse direction, hydrolysing ATP into ADP ( Figure 2). This unusual way of generating a mitochondrial potential also implies that a functional phosphate/H ^+^ exchanger must be present.

Significant differential expression of mitochondrial and glycosomal proteins occurs during the life cycle ^21^. Indeed, during the differentiation of B-LS into PFs, degradation of glycosomes probably via autophagy is enhanced and new glycosomes with different enzymatic contents are produced, so that parasites become rapidly metabolically adapted to the new host environment ^22^. In fact, the differential expression of glycosomal and mitochondrial proteins is a clear indicator of the difference in metabolic life styles between the two major life-cycle stages of *T. brucei*. **

### 3.2. Trypanosomes have specific pathways and unique enzymes

Kinetoplastida are of great intrinsic scientific interest as they have diverged very early compared with most studied eukaryotic models (for example, yeast, plants and animals), on which the foundations of molecular, biochemical and cellular biology have been built. Cytochrome oxidase (COX) is the terminal oxidase of the mammalian electron transport chain and is responsible for the reduction of oxygen to water. However, *T. brucei* possesses an additional plant-like, non-energy-conserving terminal oxidase called alternative oxidase (TAO). Indeed, B-LS forms are unique in the sense that they do not use COX but rely on TAO (step 11 in Figure 2C). TAO is 100-fold more expressed in B-LS than PFs and thus is considered a potential drug target ^23^.

Sphingolipids are a class of lipids important in cell recognition and signal transmission. To date, *T. brucei* is the only organism known to make all three types of sphingolipids (sphingomyelin, inositolphosphoceramide and ethanolamine-phosphoceramide). These lipids are synthesised via four sphingolipid synthases (SLSs) that are encoded by genes organised in a tandem array. Sphingolipid synthesis is highly controlled during development: a dedicated inositolphosphoceramide synthase (SLS1) is highly upregulated in B-SS parasites and maintained in PFs ^24^. As a consequence of more ceramide being used for inositolphosphoceramide synthesis, the synthesis of sphingomyelin is reduced, causing an alteration in the levels of phosphatidylinositol species.

## 4. Metabolic adaptations during the *Trypanosoma brucei* life cycle

The bloodstream of a mammalian host is a very rich environment, containing 5 mM of glucose, 95% to 99% oxygen saturation levels and 0.6 to 0.8 g/mL proteins, including lipoproteins (low-density lipoprotein and high-density lipoprotein). In contrast, when parasites are ingested by the tsetse during a blood meal, they end up in a glucose-poor but amino acid-rich environment that is very different from the mammalian bloodstream. Given that we can mimic these growing conditions *in vitro*, most of our knowledge about metabolic changes during the *T. brucei* life cycle originates from the comparison between B-LS and PFs.

### 4.1. Amino acids: an abundant carbon source in the midgut of the fly

The midgut of the tsetse fly has a temperature of about 28°C and a variable pH and contains hardly any glucose, but is rich in amino acids, such as proline (about 100 μM) ^9^. It is well accepted that, in a glucose-depleted environment, PFs primarily use proline for their energy production ^25, 26^ ( Figure 1), but catabolism of other amino acids, such as threonine and leucine, is also used ^27, 28^. These latter amino acids feed fatty acid biosynthesis and/or enter into the mevalonate pathway to produce the building blocks to generate essential lipids, including isoprenoids and sterols. Proline is catabolised within the mitochondrion and excreted from the cell as the end-product alanine, with the production of several reduced cofactors, which are reoxidised in the respiratory chain for ATP production by OXPHOS ( Figure 2A). However, if glucose is provided, PFs adjust their metabolism and produce most of their ATP via glucose degradation (glycolysis), even in the presence of proline ( Figure 2B) ^25, 29^. These findings highlight that these parasites, like most other eukaryotes, are extremely flexible at adapting their central metabolism to their environment.

### 4.2. Glucose: differences in consumption rate and efficiency of ATP production

So far, the only carbon source for ATP production described for replicative bloodstream parasites is glucose, which is converted via glycolysis ( Figure 1 and Figure 2C). Unlike proliferative yeast and tumour cells, B-LS does not undergo fermentation per se. In fact, instead of being metabolised and generating ethanol or lactate, most pyruvate in B-LS is immediately excreted, and only about 1% is fermented into succinate ^30, 31^. To oxidise the NADH produced during glycolysis back to NAD ^+^, B-LS consume large amounts of oxygen that act as an electron acceptor in a reaction catalysed by the unusual TAO. This type of glucose metabolism is uncommon and does not fit textbook knowledge. Interestingly, B-LS cells also tolerate anaerobic conditions where they convert glucose to equimolar amounts of glycerol and pyruvate, with a two-fold reduction of the ATP production rate.

As mentioned above, although PFs rely upon proline *in vivo*, they prefer glucose to produce ATP ^25^. Interestingly, the rate of glucose degradation is about 10-fold higher in B-LS than in PFs ^30, 32^. This considerable difference is probably due to metabolic adaptations developed by B-LS in response to a much higher ATP demand compared with PFs. Firstly, B-LS replicate faster than PFs (doubling times of about 7 and about 12 hours, respectively), which means that theoretically B-LS should show a 1.5-fold higher rate of ATP production. Secondly, the estimated number of ATP molecules produced per glucose consumed is about two-fold lower in B-LS. This difference is explained by the different strategies used by B-LS and PFs to degrade glucose into excreted end-products, which are mainly pyruvate in B-LS ( Figure 2C) and acetate plus succinate in PFs ( Figure 2B) ^33^. Indeed, at the end of glycolysis, PFs convert pyruvate into acetate and ATP by the acetate:succinate CoA-transferase (ASCT)/succinyl-CoA synthetase cycle ^34, 35^. This pathway accounting for about 70% of the glycolytic flux in PFs is reduced to 5% in B-LS ^30^. In addition, the glycosomal succinate fermentation pathway (steps 3–6 in Figure 2B), pyruvate phosphate dikinase (step 7), and cytosolic localisation of phosphoglycerate kinase (step 1b in Figure 2B) improve the rate of ATP production within the cytosol of PFs ^29, 36^.

Thirdly and probably the most important reason for a higher rate of glucose degradation in B-LS is that some biological processes require more ATP in B-LS compared with PFs. This is the case of endocytosis, which is at least about 10-fold upregulated in B-LS compared with PFs and other trypanosomatids ^37, 38^. The high endocytic activity observed in B-LS is required for rapid recycling of cell-surface glycosylphosphatidylinositol (GPI)-anchored variant surface glycoprotein (VSG) for internalisation and removal of bound antibodies, facilitating escape from the host immune defences, but also for nutrient scavenging from the mammalian host. Knockdown of actin resulted in a significant decrease (>70%) in endocytic activity and clearance of anti-VSG antibodies by B-LS forms, but did not significantly affect cellular ATP levels ^38, 39^. Surprisingly, measurement of the rates of pyruvate production and oxygen consumption, under conditions identical to those employed for the ATP and transferrin uptake assays, revealed a decrease of about four-fold in both rates after a knockdown of 15 hours (D. P. Nolan, unpublished data). Although the consumption of glucose was not measured, these data suggest that membrane trafficking in the B-LS may represent a significant additional ATP demand compared with the PFs and even more surprisingly that the rate of ATP utilisation may also influence its rate of production via glycolysis. However, knockdown of actin also led to a rapid cessation in cell division and eventual cell death, so the implications of these preliminary metabolic investigations may not be so straightforward.

Interestingly, although B-SS live in a glucose-rich environment, they undergo morphological and gene expression alterations that are consistent with a preparation to survive within the tsetse midgut environment ^7, 24^ ( Figure 1). These adaptions also include increased sensitivity to specific environmental cues that signal entry to the tsetse fly vector, as well as resistance to extracellular acidic and proteolytic stress ^40, 41^. Given that B-SS are non-proliferative and existent only in pleomorphic strains, it is more difficult to obtain large and pure quantities of this life-cycle stage *in vitro*. As a result, its metabolism has been less characterised. Nevertheless, we know that B-SS consume glucose and produce pyruvate and intermediate levels of acetate ^42^, suggesting that metabolism is being pre-adapted to the conditions in which procyclic forms will live within the tsetse midgut. Transcriptomic studies have confirmed the downregulation of several genes that encode for components of the glycosomes and are involved in glucose uptake and breakdown ^24^. Genes upregulated in B-SS include TAO, fructose-2,6- biphosphatase, specific membrane proteins, and specific lipid biosynthesic genes, including *TbSLS1* involved in inositolphosphoceramide synthesis. Further biochemical studies will be necessary to characterise and allow a better understanding of B-SS metabolism.

### 4.3. Lipids: responding to a great demand for myristate

In the bloodstream, trypanosomes are able to survive extracellularly in the mammalian host as they are coated by a dense homogenous layer of GPI-anchored VSGs. VSG coats are periodically exchanged by a mechanism of antigenic variation, protecting parasites against the host’s innate and adaptive immune responses ^43^. In B-LS forms, GPI anchors exclusively contain two myristate molecules (14 carbon fatty acid); however, myristate is present at very low levels within the mammalian bloodstream, which could not sustain the B-SL demand ^44^. Thus, it was initially thought that *de novo* synthesis of myristate occurred via a type II prokaryotic-like synthase ^45, 46^, but this synthesis is not sufficient for the GPI requirement ^47^. Hence, it has been discovered that *T. brucei* uses four microsomal elongases, with different specificities, to synthesise fatty acids in a stepwise manner ^47^. These elongases are responsible for the majority of *de novo* fatty acid synthesis in *T. brucei*. In bloodstream forms, downregulation of the elongase 3 in the pathway (which converts C14 to C18) explains the high production of myristate required for GPI anchors.

Trindade *et al*. have shown that at the RNA level, parasites in adipose tissue and blood are quite different and that many genes associated with metabolism are differentially expressed ^6^. Using pulse-chase experiments with stable isotope-labelled myristate, the authors showed that the first three enzymatic steps of fatty acid β-oxidation were active in parasites occupying the adipose tissue. In this pathway, a fatty acid molecule is converted into acetyl-CoA and a shorter fatty acid. The fate of each of these molecules remains unknown. The most ‘classic’ scenario would be that fatty acids undergo several cycles of β-oxidation, releasing acetyl-CoA, which could enter the TCA cycle, leading to the production of NADH and FADH _2_, which could result in the production of ATP by OXPHOS. This hypothesis, however, implies that complexes III and IV of the respiratory chain are active, and this has not been observed so far in *T. brucei* mammalian forms. An alternative scenario is that the fatty acid chains (normally, 16 carbon palmitate) released by adipocytes are shortened through β-oxidation to enter an anabolic process in order to produce complex lipids. The resulting acetyl-CoA could be converted into acetate and lead to the production of one ATP molecule by the action of ASCT enzyme.

*Tryponosoma cruzi* also infects adipocytes (specialised cells of adipose tissue) and is capable of consuming stored lipids ^48^. It is unclear how *T. brucei*, an extracellular parasite, accesses lipids that are stored inside adipocytes, which constitute the largest storage of lipids in a mammalian host. Inside adipocytes, the stored triglycerides can be converted via lipolysis into fatty acids and glycerol, which are eventually secreted. During a *T. brucei* infection, animals typically lose weight and serum shows hyperlipidaemia ^6, 49^. We speculate that during a *T. brucei* infection, lipolysis is increased, leading to the secretion of fatty acids, which could be readily taken up and used by parasites.

## 5. Concluding remarks and future perspectives

Fields such as immunometabolism emerged because of the development of highly sensitive metabolomic approaches, including untargeted metabolomic analysis, stable isotope labelling, mass spectrometry, and nuclear magnetic resonance ^50– 52^. Researchers discovered that during immune cell activation, the levels of many metabolites undergo alterations and these changes are directly linked to immune cell effector functions. In a way, the life cycle of a pathogen is a series of irreversible differentiation steps, in which the cells adapt to a new environment to perform new functions. In addition, the use of modern metabolomics approaches has revealed that *T. brucei* uses an incomplete TCA cycle in PFs ^53^ and that proline has a different fate in PFs depending upon high- or low-glucose availability in the medium and has allowed the identification of the carbon and nitrogen sources of essential metabolites ^53– 56^.

However, much remains to be discovered. Some questions for the future are the following:

• Besides the known glucose and proline transporters, what are the transporters of other essential nutrients?
• What is the signalling cascade that coordinates metabolism remodelling? In most eukaryotes, two key kinases are involved in nutrient sensing: target of rapamycin protein (TOR) and AMP-activated kinase (AMPK). *T. brucei*, the eukaryote with the most complex network of TOR proteins described so far, is composed of four TOR proteins ^57^, which are necessary for cell proliferation. Interestingly, knockdown of one of these proteins, TbTOR4, which appears to be kinetoplastid-specific, caused irreversible differentiation of the B-LS form into a quiescent form with properties very similar to those of the B-SS form, which suggested that TbTOR4 negatively regulates the slender-to-stumpy transition ^58^. Activation of AMPK also triggers differentiation to the quiescent B-SS forms ^59^. It is likely that some of these kinases are directly involved in remodelling metabolism during the *T. brucei* life cycle. It is interesting that changes in inositol metabolism lead to perturbations in *VSG* gene expression, suggesting that inositol metabolites are important for the control of this B-LS-specific process ^60^.
• What is the metabolism of other stages of the life cycle? With the possibility of generating *in vitro* multiple stages of tsetse life cycle by overexpressing RBP6 ^61^, it may be possible in the near future to understand metabolism of epimastigotes and metacyclics.
• Do other trypanosome species undergo similar or different metabolic adaptations during their life cycle as they encounter different environments?
• What is the metabolism of slender and stumpy forms when these occupy other tissues within the mammalian host? Trindade *et al*. have shown that at the RNA level, parasites in adipose tissue and blood are quite different and that many genes associated with metabolism are differentially expressed ^6^. It will be important in the future, perhaps for drug development, to confirm these observations at protein and metabolite levels not only in visceral adipose tissue but also in the skin and importantly in the brain.
• What are the consequences of a *T. brucei* infection in the host? Trypanosomiasis is characterised by decreased levels of aromatic amino acids, especially tryptophan, in the host and these levels are accompanied by the excretion of abnormal amounts of aromatic ketoacids, such as indole-3-pyruvate ^62^. It was recently shown that B-LS generates indole-3-pyruvate by transamination of tryptophan and secretes significant amounts of this aromatic ketoacid. Indole-3-pyruvate appears to be able to modulate the host inflammatory responses, which may prolong host survival and thereby potentiate transmission of the parasite to the tsetse fly vector and ensure completion of the life cycle ^62^. The roles of other secreted parasite metabolites that can possibly modify the host’s metabolism remain to be established.
• How does the parasite metabolism change during the day? We have recently shown that, in *T. brucei*, many genes that encode for metabolic enzymes are circadianly regulated ^63^. This cycling expression pattern leads to two peaks of intracellular ATP concentration, indicating that metabolism is indeed under circadian control. How is this control achieved? Which metabolic pathways are affected? Does this adaptation reflect the circadian variation of nutrients in the host?

The interplay between the host and pathogen and the influences upon their respective metabolisms is likely to be complex but is probably very significant since adaptation to nutrient availability is a major driving force during evolution. With the help of new and more sensitive biochemical and metabolic methodologies, it should be possible to use systems biology approaches to simultaneously characterise the metabolic changes undergone by parasites and host during an infection and within different tissues.
